# Association of blood pressure measurements in sitting, supine, and standing positions with the 10-year risk of mortality in Korean adults

**DOI:** 10.4178/epih.e2023055

**Published:** 2023-06-08

**Authors:** Inkyung Baik, Nan Hee Kim, Seong Hwan Kim, Chol Shin

**Affiliations:** 1 Department of Foods and Nutrition, Kookmin University College of Sciences and Technologies, Seoul, Korea; 2 Department of Internal Medicine, Korea University Ansan Hospital, Ansan, Korea; 3 Institute of Human Genomic Study, Korea University Ansan Hospital, Ansan, Korea

**Keywords:** Blood pressure, Posture, Hypertension, Mortality, Cardiovascular diseases

## Abstract

**OBJECTIVES:**

This prospective cohort study investigated the association between blood pressure (BP) as measured in different body postures and all-cause and cardiovascular (CV) mortality risk.

**METHODS:**

This population-based investigation included 8,901 Korean adults in 2001 and 2002. Systolic blood pressure (SBP) and diastolic blood pressure (DBP) were measured sequentially in the sitting, supine, and standing positions and classified into 4 categories: (1) normal, SBP <120 mmHg and DBP <80 mmHg; (2) high normal/prehypertension, SBP 120-129 mmHg and DBP <80 mmHg/SBP 130-139 mmHg or DBP 80-89 mmHg; (3) grade 1 hypertension (HTN), with SBP 140-159 mmHg or DBP 90-99 mmHg; and (4) grade 2 HTN, SBP ≥160 mmHg or DBP ≥100 mmHg. The date and cause of individual deaths were confirmed in the death record data compiled until 2013. Data were analyzed using Cox proportional hazard regression.

**RESULTS:**

Significant associations were found between the BP categories and all-cause mortality, but only when BPs were measured in the supine position. The multivariate hazard ratios (95% confidence intervals, [CIs]) were 1.36 (95% CI, 1.06 to 1.75) and 1.59 (95% CI, 1.06 to 2.39) for grade 1 HTN and grade 2 HTN, respectively, compared with the normal category. The associations between the BP categories and CV mortality were significant regardless of body posture among participants ≥65 years, whereas they were significant for supine BP measurements only in those <65 years.

**CONCLUSIONS:**

BP measured in the supine position predicted all-cause mortality and CV mortality better than BP measured in other postures.

## GRAPHICAL ABSTRACT

[Fig f2-epih-45-e2023055]

## INTRODUCTION

Blood pressure (BP) changes spontaneously and naturally in response to awake and sleep states, psychological and physical stress, and changes in body posture during the measurement [1,2]. To reduce variation, it is recommended that BP should be measured when the individual is seated comfortably with back support, the arm is supported at the heart level, and the feet are flat on the ground [3]. In clinical settings, however, adherence to this guideline is often neglected [4]. According to some current guidelines, BP can be measured in either the supine or sitting posture [3,5]. However, previous studies have also shown that BP varies when measured in different postures [5-9]. Therefore, posture-specific BPs and posture-specific diagnoses of hypertension (HTN) are not always consistent.

It has been reported that elevated BP or HTN increases all-cause and cardiovascular (CV) mortality risk [10-12]. However, few studies have compared the association between posture-specific BP measurement and morbidity and mortality. A recent study conducted multiple systolic blood pressure (SBP) measurements in the sitting, supine, and standing positions and compared their associations with cardiovascular disease (CVD) risk scores [13]. Meanwhile, several studies have reported on the association of BP variance due to postural changes with CV morbidity [14-16] and mortality [17-20].

The current population-based cohort study measured BPs in the sitting, supine, and standing positions, and investigated the association of posture-specific BP with the 10-year risk of all-cause mortality and CV mortality. In addition, ancillary ultrasonography was used to investigate the association between posture-specific BP and an early marker of atherosclerosis. Our goal was to determine which BP postural measurement best predicted 10-year mortality.

## MATERIALS AND METHODS

### Study population

The study participants were members of two population-based cohorts included in the Korean Genome Epidemiology Study, an ongoing longitudinal investigation. Details of the enrollment method and study procedures are available in previous reports [21,22]. Using a 2-stage cluster sampling method, we identified 10,957 Korean male and female residents aged 40 years to 69 years for enrollment in 2 cities, Ansan and Ansung. Approximately 91% of these cohorts (9,996 participants) completed on-site health examinations between June 2001 and January 2003. The examination included BP measurement, anthropometric assessment, blood collection for biochemical and genetic assays, and a questionnairebased interview. The questionnaire covered socio-demographic information, medical history and health conditions, and lifestyle factors. Similar examinations were conducted biennially during the follow-up period.

Participants who reported the use of medications for HTN or pregnancy at baseline were excluded (n= 1,095), leaving a total of 8,901 participants who were eligible for mortality follow-up. Furthermore, 3,487 participants in a sub-study initially underwent an additional examination including ultrasonography. Approximately 80% (n= 2,780) of the sub-study participants who were < 65 years old, free of diagnosed CVD at baseline, and had completed follow-up for mortality were included in the sub-study analysis.

### Determination of mortality

The date and cause of death as well as vital status were obtained from the death record data compiled by Statistics Korea, which linked each participant’s Resident Registration Number in the baseline data with the dataset of the national death registration. In this study, death record data compiled until December 31, 2013, were used (data after this date were not accessible). We were able to ascertain 99% of the death dates and 91% of the causes of death. Because the cause of death was recorded based on the International Classification of Diseases, 10th revision (ICD-10) in the original dataset, the major causes of death, such as cancer, CVD, and other diseases, were classified using ICD-10 codes.

### Measurement of carotid intima-media thickness

In the ancillary study, the intima-media thickness (IMT), which is considered an early marker of atherosclerosis [23], was measured. A detailed description of the IMT measurement method is available elsewhere [24]. All procedures for IMT measurement were performed by trained personnel who followed a standardized protocol. Using images of the right and left common carotid arteries, the carotid bifurcation, and the internal carotid artery, mean and maximum measures (≥ 1.0 mm) of the IMT were obtained at the near and far walls of four 1-cm segments proximal to the bifurcation. The final values of mean and maximum IMT were calculated by averaging the measures of the right and left sides.

### Measurement and classification of blood pressure

At baseline, BP measurement was performed by well-trained personnel who used a mercury sphygmomanometer and followed a standardized protocol for 3 postures, including sitting, supine, and standing positions. The BP was initially measured in both arms in a sitting position. To measure BP in a sitting position, the participant was instructed to sit in a chair with back support and place their feet on the floor, then to relax for 5 minutes. His or her upper arm was supported at the heart level and wrapped with the BP cuff, and repeated BP measurements were taken at ≥ 1-minute intervals in both arms. The arm with the higher value was used for subsequent measurements. To measure BP in the supine position, the participant was instructed to lie on a bed with both arms placed on the bed and to relax for 5 minutes. His or her upper arm was supported at the heart level and wrapped with the BP cuff, and repeated BP measurements were taken at ≥ 1-minute intervals. To measure BP in the standing position, the participant was instructed to stand up with his or her upper arm supported at the heart level and wrapped with the BP cuff. The first standing BP measurement was taken immediately after the postural change from a supine position, and a repeat BP measurement was taken 2 minutes after the first measurement. At least 2 measurements for each of the 3 posture positions were used to calculate the average SBP and diastolic blood pressure (DBP) values.

This study used the BP categories suggested by the Korean Society of Hypertension [25], but the high normal and prehypertension (pre-HTN) categories were combined because of the small sample size in the high normal category. Thus, 4 BP categories were defined: (1) normal, SBP < 120 mmHg and DBP < 80 mmHg; (2) high normal/pre-HTN, SBP 120-129 mmHg and DBP < 80 mmHg/SBP 130-139 mmHg or DBP 80-89 mmHg; (3) grade 1 HTN, SBP 140-159 mmHg or DBP 90-99 mmHg; and (4) grade 2 HTN, SBP ≥ 160 mmHg or DBP ≥ 100 mmHg. Thus, HTN was defined as SBP ≥ 140 mmHg or DBP ≥ 90 mmHg based on the last 2 categories.

### Confounding factors

The questionnaire inquired about age, sex, education level, smoking status, alcohol consumption, physical activity, the presence of depressive moods, diagnosis of chronic diseases, and medications for treating chronic diseases. Questions regarding smoking status and alcohol consumption included former or current tobacco use, duration of smoking, the average number of cigarettes smoked per day, the pattern and frequency of alcohol consumption during the previous 30 days, the number of drinks consumed on a typical drinking day, and the volume of a drink for each alcoholic beverage. For alcohol drinkers who consumed at least 1 drink per month, the amount of alcohol consumed (g/day) was calculated. The level of physical activity was differentiated into 5 categories of activity intensity as well as the hours spent in physical activity on a typical day. The total metabolic equivalent (MET-hr/day) score was calculated by multiplying the hours spent on a typical day by the MET values. The presence of diabetes mellitus was based on the use of insulin or hypoglycemic medications as well as blood glucose levels, which were assessed by an oral glucose tolerance test. Height (cm) and body weight (kg) were measured to calculate body mass index (BMI, kg/m^2^).

### Statistical analysis

Descriptive statistics regarding the baseline characteristics of the study population were calculated according to the 4 BP classifications. The degree of agreement (kappa statistics) was calculated for HTN according to 2 different body postures.

To analyze the associations between BP classification and the 10-year risk of all-cause and cause-specific mortality, we used Cox proportional hazards regression and the Efron approximation method to obtain hazard ratios (HRs) and 95% confidence intervals (CIs). The person-years were calculated from the date of an individual’s baseline examination, to the date of his or her death during the follow-up period or to the last date of follow-up (December 31, 2013), whichever came first. In multivariate models, age and BMI were fitted as continuous variables while sex, education level (≤9 or >9 years), smoking status (never, formerly smoked, smoking ≤ 10, 11-20, or > 20 cigarette/day), alcohol consumption (none, < 15, 15-30, or > 30 g/day), physical activity (quintile of MET-hr/day), depressive moods (no or yes), diabetes mellitus (no or yes), and diagnosed cancer or CVD (no or yes) were fitted as categorical variables. When the proportional hazards assumptions were tested for a full model that included all potential confounding factors, no violation was confirmed.

To analyze the associations between BP categories and mean IMT scores, linear regression analysis was conducted, and regression coefficient estimates (95% CI) were obtained. Multiple models included the same confounding variables described above. SAS version 9.4 (SAS Institute Inc., Cary, NC, USA) was used to conduct all tests based on a 2-sided level of significance.

### Ethics statement

All procedures and protocols of the baseline and follow-up examinations were standardized and approved by the Human Subjects Review committees of the 2 study sites (Korea University Ansan Hospital and Ajou University Medical Center), and participants signed an informed consent form during each visit (IRB No. ED0624 and AJIRB-MED-OBS-16-509).

## RESULTS

### Characteristics of the study participants

The mean age of the 8,901 participants (4,327 male and 4,574 female) was 51.6± 8.8 years. The mean length of the follow-up period was 10.0 ± 1.5 years and 528 deaths were documented. The major causes of death were cancer (n= 231), CVD (n= 93), and other diseases such as liver disease (n= 19), diabetes mellitus (n= 14), and chronic lower respiratory disease (n= 12).

The kappa values between postural HTN and non-HTN were 0.55 for sitting and supine positions, 0.59 for sitting and standing positions, and 0.54 for supine and standing positions. Descriptive statistics of the posture-specific BP measurements and weighted kappa statistics are presented in the Supplementary Material 1. Kappa values for SBP were higher than those for DBP.

Descriptive statistics of the demographic and clinical characteristics and the lifestyle factors according to BP classification are presented in Table 1. Participants in the HTN categories showed higher mortality than those in the normal and high normal/pre-HTN categories. They were more likely to be older, male, less educated, heavier, current alcohol drinkers, and diagnosed with diabetes mellitus. Within the same category, the mean SBP and DBP measured in the supine position were likely to be lower than those measured in the sitting and standing positions. Similarly, the mean SBP and DBP measured in the standing position were likely to be lower than those measured in the sitting position in all but the normal category.

### Association between blood pressure and mortality

In Table 2, the HRs (95% CIs) for the association between each BP category and the 10-year risk of all-cause mortality are presented according to body position. Only in the multivariate models with BP measured in the supine position did participants who had HTN show a significantly elevated risk of all-cause mortality compared with those in the normal category. The results remain consistent after controlling for a diagnosis of cancer or CVD.

The multivariate results for the association between BP categories and the 10-year risk of CV mortality according to sitting, supine, and standing positions are demonstrated in Table 3. Regardless of body posture, participants in the HTN categories showed a significantly elevated risk of CV mortality compared with those in the normal category. The HR for the supine position was higher than for the sitting and standing positions. In the age-specific analysis, those ≥ 65 years showed associations like those observed among the participants overall. However, for those < 65 years, a significant association was observed only in the supine position; the HRs (95% CIs) were 2.25 (95% CI, 1.10 to 4.59) for grade 1 HTN and 3.55 (95% CI, 1.29 to 9.77) for grade 2 HTN when compared with the normal category. When analyzing similar data for mortality due to cancer or other diseases, no significant associations were observed (Supplementary Material 2).

**Figure 1.**
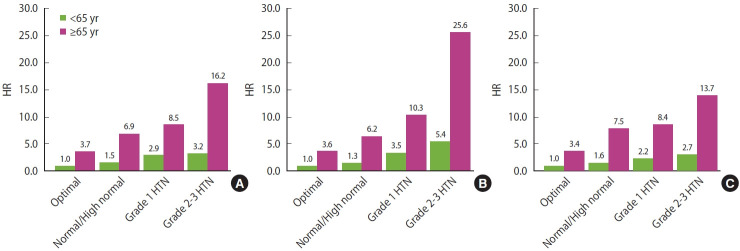
HRs for the joint analysis of age groups (<65 and ≥65 years) and blood pressure categories in association with cardiovascular mortality. Blood pressure categories (X axis): normal, SBP <120 mmHg and DBP <80 mmHg; high normal/prehypertension, SBP 120-129 mmHg and DBP <80 mmHg/SBP 130-139 mmHg or DBP 80-89 mmHg; grade 1 HTN, SBP 140-159 mmHg or DBP 90-99 mmHg; grade 2 HTN, SBP ≥160 mmHg or DBP ≥100 mmHg. The results are shown for sitting (A), supine (B), and standing (C) positions. HRs (Y axis) were estimated after data were adjusted for sex, education level (≤9 or >9 years), body mass index, smoking status (never smoked, former smoker, smokes ≤10, 11-20, or >20 cigarette/day), alcohol consumption (none, <15, 15-30, or >30 g/day), physical activity (quintiles of MET-hr/day), depressive moods (no or yes), and diabetes mellitus (no or yes). HR, hazard ratio; SBP, systolic blood pressure; DBP, diastolic blood pressure; HTN, hypertension; MET, metabolic equivalent.

The multivariate results for the joint analysis of BP categories and age groups in relation to CV mortality when BP was measured in the sitting, supine, and standing positions are shown in Figure 1. For each position, a reference group was set that included the younger participants in the optimal category. The HRs for the HTN categories in the B panel were higher than those in the A panel and C panel for both age groups. When compared with the reference group, the HR (95% CI) was 3.45 (95% CI, 1.71 to 6.99) for grade 1 HTN and 5.36 (95% CI, 1.98 to 14.50) for grade 2 HTN among participants aged < 65 years, while it was 10.32 (95% CI, 4.84 to 22.01) for grade 1 HTN and 25.60 (95% CI, 9.99 to 65.81) for grade 2 HTN among older participants.

Additional analyses were conducted to evaluate whether the BP differences in different postures were associated with CV mortality. To obtain the differences in SBP (∆SBP) and DBP (∆DBP), the supine BP was subtracted from the sitting BP, the supine BP was subtracted from the standing BP, or the standing BP was subtracted from the sitting BP. The ∆SBP and ∆DBP results were classified into 2 groups, < 5 mmHg and ≥ 5 mmHg, and their associations with CV mortality were analyzed. Only the ∆SBP between sitting SBP and supine SBP was significantly associated with CV mortality; compared with the group with ∆SBP ≥ 5 mmHg, the HR (95% CI) for the group of ∆SBP < 5 mmHg was 1.56 (95% CI, 1.02 to 2.40). No significant association was observed in the ∆SBP and ∆DBP between other postures (Supplementary Material 3).

### Association between blood pressure and carotid intima-media thickness

To explore a potential explanation for the association between HTN and CV mortality, we investigated the association between the BP categories and carotid IMT among participants < 65 years who were free of diagnosed CVD at baseline. The results of linear regression analysis for IMT scores are presented in Table 4. Only when BP was measured in the supine position was there a significant association between the grade 2 HTN category and mean IMT scores (p< 0.05); the mean IMT value was 0.71 mm in the normal BP category and 0.73 mm in those with grade 2 HTN. However, no associations between BP classification and IMT were observed when the BP was measured in a sitting or standing position. Among those aged ≥ 65 years, no significant result was observed (Supplementatary Material 4).

## DISCUSSION

In the present study, we attempted BP measurement in 3 postures (sitting, supine, and standing) to investigate the association with 10-year mortality. We observed that HTN based on a supine BP had a stronger association with all-cause mortality and CV mortality than BP measured in sitting or standing positions. Among younger participants, we found a significant association between HTN and CV mortality only when BP was measured in the supine position. In an ancillary ultrasonography study including younger participants who were free of diagnosed CVD, we also found a significant association between HTN based on a supine BP and carotid IMT, but there was no significant association with other body positions.

Several studies have observed BP variations according to body position [5-9]. A population-based study observed that higher SBPs and DBPs were measured in the sitting position than in the supine position [8]. This result is consistent with our findings, but differs from other studies that reported higher supine SBP than sitting SBP [5,6,9] or higher supine DBP than sitting DBP [7,9] in patients with HTN or diabetes. Although these discrepancies may be partly explained by the different BP measurement protocols, the diverse demographic characteristics and disease status of the study subjects, or the different sample sizes, these are not complete explanations. A major concern regarding the variation in BP according to body posture is the potential for misclassification when diagnosing HTN if the body posture is not considered in BP measurement [7]. Although the accuracy of BP measurement has been emphasized in diagnosing HTN [3], both sitting and supine positions have been allowed in guidelines [3]. In this study, the kappa values calculated for the non-HTN and HTN categories for 2 different body postures ranged between 0.5 and 0.6. Based on this degree of agreement, the body position needs to be specified when the BP is measured, and a posture-specific BP classification should be used exclusively.

Krzesiński et al. [26] estimated the sensitivity and specificity of BP measured in the sitting and supine positions using 24-hour ambulatory BP monitoring as the gold standard method. They found that the sensitivity and specificity estimates for supine BP measurements were higher than those for sitting BP measurements when diagnosing HTN based on the gold standard method, suggesting that a supine BP better reflects the nighttime BP as well as the daytime BP [26]. Furthermore, a diagnosis of HTN based on the supine BP has been associated with carotid IMT [27] and supine SBP measurements have been associated with CV mortality [28]. To the best of our knowledge, however, a comparison of the association between BP values measured in different postures and mortality has not been reported. However, several studies have focused on the associations between postural BP changes, specifically BP changes between standing and supine positions, and total and CV morbidity [14-20]. A recent study of elderly Chinese adults reported a significant association between total and CV mortality and heart rate measured in the supine but not sitting position [29].

The present study compared associations between posturespecific BP measurements and mortality risk. We observed a significantly elevated risk of all-cause mortality in HTN with SBP ≥ 140 mmHg or DBP ≥ 90 mmHg only when the BP was measured in a supine position. This appears to be due to the stronger association of a supine BP with CV mortality than for other postures. In particular, HTN could significantly predict CV mortality among younger participants, but only when the diagnosis is based on a supine BP. Based on these findings, elevated BP in the supine position better reflects the presence of early atherosclerosis among younger participants than that measured in other postures. In fact, our ancillary study, which revealed a significant association between supine BP and carotid IMT in younger participants who were free of diagnosed CVD, upholds this postulation. Several potential explanations support the hypothesis that supine BP measurements better predict atherosclerosis than sitting or standing BP: (1) a more accurate BP measurement is obtained in the supine position because the subject is more relaxed; (2) a supine BP better reflects nighttime BP, which is important because an elevated BP at night has been associated with CV mortality [30] and carotid atherosclerosis [31]; and (3) based on our findings that BP differences from a sitting to supine position < 5 mmHg increased the risk of CV mortality, an elevated BP in the supine position may be directly linked to vascular damage [27].

The strengths of our study include the prospective observation of a population-based cohort, a high rate of outcome ascertainment, and a broad range of information on confounding factors. In addition, our study yielded findings applicable in clinical settings to predict CV mortality. The study limitations included not being able to exclude the effects of one posture on the subsequent posture. BP values in the standing position might be influenced by postural changes, particularly among participants with orthostatic hypotension. There may be errors in BP measurement leading to null associations even though the trained personnel followed a standardized protocol.

In addition, we could only obtain the cohort members’ death record data up to 2013. The generalizability of our findings was also limited in terms of age and ethnicity.

In summary, this population-based prospective cohort study observed significant associations between BP measured in the supine position and the 10-year risk of all-cause and CV mortality, as well as atherosclerotic morbidity. These associations were found to be stronger than those for measurements made in sitting and standing positions, especially for participants aged 40 years to 64 years. Based on our findings, we suggest that BP measurements in a supine position may be more useful in assessing the risks of CV mortality and morbidity for middle-aged people than BP measurements in a sitting or standing position.

## Figures and Tables

**Figure f2-epih-45-e2023055:**
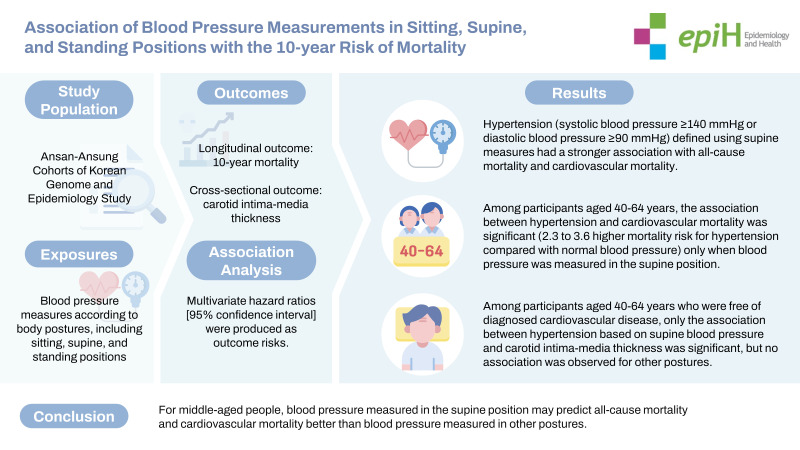


**Table 1.** Baseline characteristics of 8,901 study participants according to 4 blood pressure categories^1^

Characteristics	All participants	Categories of blood pressure				p for trend
		Normal	High normal/pre-HTN	Grade 1 HTN	Grade 2 HTN	
Participants (total)	8,901	3,915 (44.0)	3,008 (33.8)	1,463 (16.4)	515 (5.8)	
Deaths	528 (5.9)	175 (4.5)	180 (6.0)	128 (8.8)	45 (8.7)	<0.001
Sitting SBP (mmHg)	119.6±17.4	105.6±8.2	122.8±7.6	137.9±10.2	156.3±15.2	<0.001
Sitting DBP (mmHg)	79.4±11.1	70.2±6.0	81.7±4.9	91.3±5.2	102.2±7.8	<0.001
Supine SBP (mmHg)	115.7±17.2	104.3±10.3	117.7±11.3	131.2±13.8	147.3±17.6	<0.001
Supine DBP (mmHg)	74.3±11.2	67.4±8.4	75.6±8.1	83.4±8.6	92.3±10.7	<0.001
Standing SBP (mmHg)	116.8±16.6	106.0±10.2	118.7±11.2	131.0±13.4	147.5±17.1	<0.001
Standing DBP (mmHg)	79.7±11.5	72.4±8.3	81.2±8.5	89.2±8.0	99.2±10.0	<0.001
Age (yr)	51.6±8.8	49.1±8.0	52.8±8.9	54.7±8.7	54.9±8.8	<0.001
Male	4,327 (48.6)	1,631 (41.7)	1,620 (53.9)	797 (54.5)	279 (54.1)	<0.001
Education >9 yr	3,947 (44.3)	2,047 (52.3)	1,240 (41.2)	496 (33.9)	164 (31.8)	<0.001
Body mass index (kg/m^2^)	24.4±3.1	23.8±2.9	24.6±3.1	25.1±3.2	25.6±3.3	<0.001
Current smokers	2,373 (26.7)	985 (25.2)	852 (28.3)	404 (27.6)	132 (25.6)	0.098
Current alcohol drinkers	4,378 (49.2)	1,791 (45.8)	1,549 (51.5)	766 (52.4)	272 (52.8)	<0.001
Physical activity (MET-hr/day)	31.2±15.5	29.2±14.2	32.2±15.9	33.6±16.8	33.6±16.7	<0.001
Depressive mood	2,583 (29.0)	1176 (30.0)	840 (27.9)	423 (28.9)	144 (28.0)	0.174
Diabetes mellitus	977 (11.0)	310 (7.9)	359 (11.9)	225 (15.4)	83 (16.1)	<0.001

Values are presented as mean±standard deviation or number (%). pre-HTN, prehypertension; HTN, hypertension; SBP, systolic blood pressure; DBP, diastolic blood pressure; MET, metabolic equivalent.

1 Blood pressure categories, based on measurement in a sitting position: normal, SBP <120 mmHg and DBP <80 mmHg; high normal/pre-HTN, SBP 120-129 mmHg and DBP <80 mmHg/SBP 130-139 mmHg or DBP 80-89 mmHg; grade 1 HTN, SBP 140-159 mmHg or DBP 90-99 mmHg; grade 2 HTN, SBP ≥160 mmHg or DBP ≥100 mmHg.

**Table 2.** Hazard ratios for the association of blood pressure measured in the sitting, supine, and standing positions with the 10-year risk of all-cause mortality (n=8,901 participants)

Positions	Blood pressure categories^1^	No. of deaths	Person-years	All-cause mortality^2^		
				Age-adjusted model	Multivariate model 1	Multivariate model 2
Sitting	Normal	175/3,915	39,563.9	1.00 (reference)	1.00 (reference)	1.00 (reference)
	High normal/pre-HTN	180/3,008	30,150.0	0.91 (0.74, 1.13)	0.89 (0.72, 1.10)	0.89 (0.72, 1.11)
	Grade 1 HTN	128/1,463	14,465.4	1.15 (0.91, 1.45)	1.18 (0.93, 1.49)	1.18 (0.93, 1.50)
	Grade 2 HTN	45/515	5,078.5	1.13 (0.81, 1.57)	1.18 (0.84, 1.65)	1.18 (0.84, 1.65)
Supine	Normal	236/5,243	52,953.4	1.00 (reference)	1.00 (reference)	1.00 (reference)
	High normal/pre-HTN	171/2,479	24,748.6	0.99 (0.81, 1.21)	0.99 (0.81, 1.21)	0.99 (0.81, 1.21)
	Grade 1 HTN	94/945	9,271.6	1.30 (1.02, 1.67)	1.35 (1.06, 1.74)	1.36 (1.06, 1.75)
	Grade 2 HTN	27/234	2,284.2	1.47 (0.98, 2.19)	1.59 (1.06, 2.40)	1.59 (1.06, 2.39)
Standing	Normal	190/3,975	40,111.5	1.00 (reference)	1.00 (reference)	1.00 (reference)
	High normal/pre-HTN	194/2,995	29,982.7	1.07 (0.88, 1.31)	1.07 (0.87, 1.31)	1.07 (0.88, 1.31)
	Grade 1 HTN	106/1,410	13,985.5	1.14 (0.90, 1.45)	1.20 (0.94, 1.54)	1.21 (0.94, 1.54)
	Grade 2 HTN	38/521	5,178.2	1.12 (0.79, 1.59)	1.21 (0.85, 1.73)	1.21 (0.85, 1.72)

Values are presented as hazard ratio (95% confidence interval). pre-HTN, prehypertension; HTN, hypertension; SBP, systolic blood pressure; DBP, diastolic blood pressure; MET, metabolic equivalent.

1 Blood pressure categories based on measurement in a sitting position: normal, SBP <120 mmHg and DBP <80 mmHg; high normal/prehypertension, SBP 120-129 mmHg and DBP <80 mmHg/SBP 130-139 mmHg or DBP 80-89 mmHg; grade 1 HTN, SBP 140-159 mmHg or DBP 90-99 mmHg; grade 2 HTN, SBP ≥160 mmHg or DBP ≥100 mmHg.

2 Multivariate model 1: adjusted for age, sex, education level (≤9 or >9 years), body mass index, smoking status (never smoked, former smoker, smokes ≤10, 11-20, or >20 cigarette/day), alcohol consumption (none, <15, 15-30, or >30 g/day), physical activity (quintiles of MET-hr/day), depressive mood (no or yes), and diabetes mellitus (no or yes); Multivariate model 2: adjusted for the covariates of multivariate model 1 plus the presence of diagnosed cancer or cardiovascular disease (no or yes).

**Table 3.** Hazard ratio for the association of blood pressure measured in the sitting, supine, and standing positions with the 10-year risk of cardiovascular mortality^1^

Positions	Blood pressure categories^2^	No. of deaths	Cardiovascular mortality		
			All (n=8,901)	<65 yr (n=7,877)	≥65 yr (n=1,024)
Sitting	Normal	19/3,915	1.00 (reference)	1.00 (reference)	1.00 (reference)
	High normal/pre-HTN	33/3,008	1.33 (0.75, 2.37)	1.10 (0.54, 2.27)	1.99 (0.72, 5.48)
	Grade 1 HTN	27/1,463	1.98 (1.08, 3.64)	1.95 (0.92, 4.14)	2.33 (0.81, 6.72)
	Grade 2 HTN	14/515	2.79 (1.36, 5.71)	2.09 (0.78, 5.63)	4.56 (1.44, 14.45)
Supine	Normal	29/5,243	1.00 (reference)	1.00 (reference)	1.00 (reference)
	High normal/pre-HTN	29/2,479	1.20 (0.71, 2.03)	0.93 (0.46, 1.88)	1.75 (0.74, 4.14)
	Grade 1 HTN	24/945	2.38 (1.36, 4.16)	2.25 (1.10, 4.59)	2.91 (1.14, 7.43)
	Grade 2 HTN	11/234	4.72 (2.29, 9.73)	3.55 (1.29, 9.77)	7.97 (2.64, 24.09)
Standing	Normal	22/3,975	1.00 (reference)	1.00 (reference)	1.00 (reference)
	High normal/pre-HTN	37/2,995	1.58 (0.92, 2.69)	1.26 (0.63, 2.50)	2.19 (0.90, 5.31)
	Grade 1 HTN	22/1,410	1.95 (1.06, 3.57)	1.79 (0.82, 3.90)	2.29 (0.85, 6.17)
	Grade 2 HTN	12/521	2.64 (1.29, 5.43)	2.16 (0.82, 5.73)	3.89 (1.26, 12.05)

Values are presented as hazard ratio (95% confidence interval). pre-HTN, prehypertension; HTN, hypertension; MET, metabolic equivalent; SBP, systolic blood pressure; DBP, diastolic blood pressure.

1 Data were adjusted for age, sex, education level (≤9 or >9 years), body mass index, smoking status (never smoked, former smoker, smokes ≤10, 11-20, or >20 cigarette/day), alcohol consumption (none, <15, 15-30, or >30 g/day), physical activity (quintiles of MET-hr/day), depressive moods (no or yes), and diabetes mellitus (no or yes).

2 Blood pressure categories based on measurement in a sitting position: normal, SBP <120 mmHg and DBP <80 mmHg; high normal/prehypertension, SBP 120-129 mmHg and DBP <80 mmHg/SBP 130-139 mmHg or DBP 80-89 mmHg; grade 1 HTN, SBP 140-159 mmHg or DBP 90-99 mmHg; grade 2 HTN, SBP ≥160 mmHg or DBP ≥100 mmHg.

**Table 4.** Association of blood pressure measured in the sitting, supine, and standing positions with carotid IMT in 2,780 participants younger than 65 years who were free of diagnosed cardiovascular disease

Positions	Blood pressure categories^1^	No. of participants	Mean IMT scores^2^ mean±SD	Coefficient estimates (95% CI) for mean IMT scores^2^	
				Age-adjusted model	Multiple model^3^
Sitting	Normal	1,060	70.9±7.9	1.00 (reference)	1.00 (reference)
	High normal/pre-HTN	1,017	71.2±8.0	0.23 (-0.46, 0.91)	0.21 (-0.48, 0.91)
	Grade 1 HTN	524	70.8±7.8	-0.10 (-0.95, 0.74)	-0.11 (-0.98, 0.75)
	Grade 2 HTN	179	71.7±8.1	0.78 (-0.49, 2.04)	0.73 (-0.57, 2.02)
Supine	Normal	1,499	70.9±8.0	1.00 (reference)	1.00 (reference)
	High normal/pre-HTN	871	71.1±7.9	0.21 (-0.46, 0.89)	0.21 (-0.48, 0.89)
	Grade 1 HTN	326	70.8±7.7	-0.09 (-1.05, 0.88)	-0.10 (-1.08, 0.89)
	Grade 2 HTN	84	72.9±8.1	2.00 (0.25, 3.74)^*^	1.96 (0.19, 3.73)^*^
Standing	Normal	1,100	70.9±8.0	1.00 (reference)	1.00 (reference)
	High normal/pre-HTN	987	70.9±7.8	0.01 (-0.68, 0.69)	0.01 (-0.69, 0.70)
	Grade 1 HTN	493	71.6±7.9	0.73 (-0.11, 1.58)	0.76 (-0.11, 1.63)
	Grade 2 HTN	200	71.3±8.0	0.38 (-0.82, 1.57)	0.39 (-0.84, 1.61)

IMT, intima-media thickness; SD, standard deviation; CI, confidence interval; pre-HTN, prehypertension; HTN, hypertension; MET, metabolic equivalent; SBP, systolic blood pressure; DBP, diastolic blood pressure.

1 Blood pressure categories based on measurement in a sitting position: normal, SBP <120 mmHg and DBP <80 mmHg; high normal/pre-HTN, SBP 120-129 mmHg and DBP <80 mmHg/SBP 130-139 mmHg or DBP 80-89 mmHg; grade 1 HTN, SBP 140-159 mmHg or DBP 90-99 mmHg; grade 2 HTN, SBP ≥160 mmHg or DBP ≥100 mmHg.

2 Raw data of mean IMT scores were multiplied by 100.

3 In the multivariate model, data were adjusted for age, sex, education level (≤9 or >9 years), body mass index, smoking status (never smoked, former smoker, smokes ≤10, 11-20, or >20 cigarette/day), alcohol consumption (none, <15, 15-30, or >30 g/day), physical activity (quintiles of MET-hr/day), depressive moods (no or yes), and diabetes mellitus (no or yes).

* p<0.05.

## SUPPLEMENTARY MATERIALS

Supplementary Material 1. Descriptive statistics of posture-specific blood pressure measures and weighted kappa statistics

Supplementary Material 2 Hazard ratio for the association of blood pressure measured in the sitting, supine, and standing positions with the 10-year risk of mortality due to cancer and other diseases

Supplementary Material 3 Hazard ratio for the association between differences in postural blood pressure and the 10-year risk of cardiovascular mortality

Supplementary Material 4 Association of blood pressure measured in the sitting, supine, and standing positions with carotid intima-media thickness in 592 participants aged 65 years or older who were free of diagnosed cardiovascular disease
