# Supplementary material for: Association of blood pressure measurements in sitting, supine, and standing positions with the 10-year risk of mortality in Korean adults
Source: Epidemiol Health. 2023 Jun 8;45:e2023055. doi: 10.4178/epih.e2023055 (PMC10482565; doi:10.4178/epih.e2023055)
Supplement: Supplementary Material 1. — Descriptive statistics of posture-specific blood pressure measures and weighted kappa statistics [file epih-45-e2023055-Supplementary-1.docx]

Supplementary Material 1. Descriptive statistics of posture-specific blood pressure measures and weighted kappa statistics

|  | Weighted kappa | Blood pressure classification | | | |
| --- | --- | --- | --- | --- | --- |
|  | for 4×4 strata | Normal | High normal/pre-HTN | Grade 1 HTN | Grade 2-3 HTN |
| SBP |  |  |  |  |  |
| Sitting position, mean±S.D. |  | 105.6±8.1 | 119.4±6.0 | 127.1±7.3 | 142.7±14.2 |
| Supine position, mean±S.D. |  | 104.8±9.1 | 121.7±5.2 | 130.3±6.2 | 145.3±13.6 |
| Standing position, mean±S.D. |  | 103.9±8.3 | 117.5±7.1 | 124.0±8.5 | 137.8±14.6 |
| Sitting vs supine strata | 0.63 |  |  |  |  |
| Sitting vs standing strata | 0.60 |  |  |  |  |
| Supine vs standing strata | 0.65 |  |  |  |  |
| DBP |  |  |  |  |  |
| Sitting position, mean±S.D. |  | 70.2±6.0 | 79.4±4.0 | 84.6±4.5 | 94.1±7.7 |
| Supine position, mean±S.D. |  | 67.8±7.7 | 78.1±5.2 | 83.0±5.7 | 91.4±8.3 |
| Standing position, mean±S.D. |  | 70.2±6.3 | 79.6±4.3 | 85.4±3.7 | 95.3±7.4 |
| Sitting vs supine strata | 0.45 |  |  |  |  |
| Sitting vs standing strata | 0.54 |  |  |  |  |
| Supine vs standing strata | 0.47 |  |  |  |  |

Abbreviations: pre-HTN, prehypertension; HTN, hypertension; SBP, systolic blood pressure; DBP, diastolic blood pressure
