# Supplementary material for: Association of blood pressure measurements in sitting, supine, and standing positions with the 10-year risk of mortality in Korean adults
Source: Epidemiol Health. 2023 Jun 8;45:e2023055. doi: 10.4178/epih.e2023055 (PMC10482565; doi:10.4178/epih.e2023055)
Supplement: Supplementary Material 2 — Hazard ratio for the association of blood pressure measured in the sitting, supine, and standing positions with the 10-year risk of mortality due to cancer and other diseases [file epih-45-e2023055-Supplementary-2.docx]

Supplementary Material 2. Hazard ratio for the association of blood pressure measured in the sitting, supine, and standing positions with the 10-year risk of mortality due to cancer and other diseases

| Positions | Blood pressure | Number of | HR (95% CI) | Number of | | HR (95% CI) |
| --- | --- | --- | --- | --- | --- | --- |
|  | classification^a^ | deaths/participants | Cancer | | deaths/participants | Other diseases |
| Sitting | Normal | 82/3915 | reference | | 33/3915 | reference |
|  | High normal/pre-HTN | 75/3008 | 0.82 (0.59, 1.12) | | 43/3008 | 1.10 (0.69, 1.75) |
|  | Grade 1 HTN | 60/1463 | 1.23 (0.87, 1.74) | | 21/1463 | 0.95 (0.54, 1.68) |
|  | Grade 2 HTN | 14/515 | 0.81 (0.46, 1.46) | | 7/515 | 0.83 (0.36, 1.92) |
|  |  |  |  | |  |  |
| Supine | Normal | 112/5243 | reference | | 46/5243 | reference |
|  | High normal/pre-HTN | 76/2479 | 0.93 (0.69, 1.26) | | 37/2479 | 1.00 (0.64, 1.56) |
|  | Grade 1 HTN | 36/945 | 1.10 (0.75, 1.62) | | 16/945 | 1.00 (0.56, 1.80) |
|  | Grade 2 HTN | 7/234 | 0.92 (0.43, 2.00) | | 5/234 | 1.15 (0.44, 2.97) |
|  |  |  |  | |  |  |
| Standing | Normal | 88/3975 | reference | | 39/3975 | reference |
|  | High normal/pre-HTN | 85/2995 | 1.05 (0.77, 1.42) | | 42/2995 | 1.08 (0.69, 1.68) |
|  | Grade 1 HTN | 43/1410 | 1.08 (0.74, 1.58) | | 21/1410 | 1.12 (0.65, 1.93) |
|  | Grade 2 HTN | 15/521 | 1.15 (0.66, 2.01) | | 2/521 | 0.29 (0.07, 1.22) |

Abbreviations: pre-HTN, prehypertension; HTN, hypertension; HR, hazard ratio; CI, confidence interval

Data were adjusted for age, sex, educational level (≤ 9 years or > 9 years), body mass index, smoking status (never smoked, formerly smoked, smoking ≤ 10 cigarettes/day, 11-20 cigarettes/day, or > 20 cigarettes/day), alcohol drinking status (abstained, consumption of alcohol < 15g/day, 15-30g/day, or > 30g/day), physical activity (quintiles of MET-hours/day), having depressive moods (no or yes), and presence of diabetes mellitus (no or yes).

^a^Its definition was indicated in table 1.
