# Supplementary material for: Association of blood pressure measurements in sitting, supine, and standing positions with the 10-year risk of mortality in Korean adults
Source: Epidemiol Health. 2023 Jun 8;45:e2023055. doi: 10.4178/epih.e2023055 (PMC10482565; doi:10.4178/epih.e2023055)
Supplement: Supplementary Material 3 — Hazard ratio for the association between differences in postural blood pressure and the 10-year risk of cardiovascular mortality [file epih-45-e2023055-Supplementary-3.docx]

Supplementary Material 3. Hazard ratio for the association between differences in postural blood pressure and the 10-year risk of cardiovascular mortality

|  |  | Number of | CVD mortality |
| --- | --- | --- | --- |
|  | Difference | deaths/participants | Multivariate HR (95% CI) |
| Sitting SBP - Supine SBP | <5 mmHg | 62/5009 | 1.56 (1.01, 2.40) |
|  | ≥5 mmHg | 31/3892 | reference |
|  |  |  |  |
| Sitting DBP - Supine DBP | <5 mmHg | 48/4511 | 1.05 (0.70, 1.58) |
|  | ≥5 mmHg | 45/4390 | reference |
|  |  |  |  |
| Standing SBP - Supine SBP | <5 mmHg | 73/5992 | 1.26 (0.76, 2.09) |
|  | ≥5 mmHg | 20/2909 | reference |
|  |  |  |  |
| Standing DBP - Supine DBP | ≤-5 mmHg | 54/4349 | 1.24 (0.82, 1.88) |
|  | ≥-5 mmHg | 39/4552 | reference |
|  |  |  |  |
| Sitting SBP - Standing SBP | <5 mmHg | 44/5084 | 0.99 (0.66, 1.50) |
|  | ≥5 mmHg | 49/3817 | reference |
|  |  |  |  |
| Sitting DBP - Standing DBP | ≤-5 mmHg | 69/6498 | 1.29 (0.81, 2.06) |
|  | ≥-5 mmHg | 24/2403 | reference |

Abbreviations: SBP, systolic blood pressure; DBP, diastolic blood pressure; CVD, cardiovascular disease; HR, hazard ratio; CI, confidence interval

Data were adjusted for age, sex, educational level (≤ 9 years or > 9 years), body mass index, smoking status (never smoked, formerly smoked, smoking ≤ 10 cigarettes/day, 11-20 cigarettes/day, or > 20 cigarettes/day), alcohol drinking status (abstained, consumption of alcohol < 15g/day, 15-30g/day, or > 30g/day), physical activity (quintiles of MET-hours/day), having depressive moods (no or yes), and presence of diabetes mellitus (no or yes).
