# Supplementary material for: Association of blood pressure measurements in sitting, supine, and standing positions with the 10-year risk of mortality in Korean adults
Source: Epidemiol Health. 2023 Jun 8;45:e2023055. doi: 10.4178/epih.e2023055 (PMC10482565; doi:10.4178/epih.e2023055)
Supplement: Supplementary Material 4 — Association of blood pressure measured in the sitting, supine, and standing positions with carotid intima-media thickness in 592 participants aged 65 years or older who were free of diagnosed cardiovascular disease [file epih-45-e2023055-Supplementary-4.docx]

Supplementary Material 4. Association of blood pressure measured in the sitting, supine, and standing positions with carotid intima-media thickness in 592 participants aged 65 years or older who were free of diagnosed cardiovascular disease

| Positions | Blood pressure | Number | Mean IMT scores^b^ | Coefficient estimates (95% CI) for mean IMT scores^b^ | |
| --- | --- | --- | --- | --- | --- |
|  | classification^a^ | of participants | Mean (SD) | Age-adjusted model | Multiple model |
| Sitting | Normal | 126 | 71.1 (8.0) | reference | reference |
|  | High normal/pre-HTN | 245 | 71.1 (7.4) | 0.04 (-1.59, 1.67) | 0.09 (-1.55, 1.73) |
|  | Grade 1 HTN | 157 | 71.8 (7.2) | 0.77 (-1.00, 2.55) | 0.84 (-0.96, 2.64) |
|  | Grade 2 HTN | 64 | 70.5 (8.1) | -0.57 (-2.86, 1.71) | -0.32 (-2.64, 2.01) |
|  |  |  |  |  |  |
| Supine | Normal | 191 | 70.5 (7.8) | reference | reference |
|  | High normal/pre-HTN | 251 | 71.5 (7.6) | 1.04 (-0.38, 2.46) | 1.12 (-0.31, 2.55) |
|  | Grade 1 HTN | 119 | 71.4 (7.1) | 0.97 (-0.77, 2.71) | 1.05 (-0.70, 2.80) |
|  | Grade 2 HTN | 31 | 72.9 (7.8) | 2.39 (-0.48, 5.26) | 2.74 (-0.17, 5.64) |
|  |  |  |  |  |  |
| Standing | Normal | 178 | 71.2 (7.9) | reference | reference |
|  | High normal/pre-HTN | 232 | 71.0 (7.5) | -0.19 (-1.67, 1.29) | 0.06 (-1.44, 1.55) |
|  | Grade 1 HTN | 132 | 71.8 (7.5) | 0.63 (-1.08, 2.34) | 0.87 (-0.88, 2.63) |
|  | Grade 2 HTN | 50 | 71.0 (7.1) | -0.10 (-2.49, 2.28) | 0.29 (-2.14, 2.73) |

Abbreviations: CI, confidence interval; IMT, intima-media thickness; pre-HTN, prehypertension; HTN, hypertension

In the multivariate model, data were adjusted for age, sex, educational level (≤ 9 years or > 9 years), body mass index, smoking status (never smoked, formerly smoked, smoking ≤ 10 cigarettes/day, 11-20 cigarettes/day, or > 20 cigarettes/day), alcohol drinking status (abstained, consumption of alcohol < 15g/day, 15-30g/day, or > 30g/day), physical activity (quintiles of MET-hours/day), having depressive moods (no or yes), and presence of diabetes mellitus (no or yes).

^a^Its definition was indicated in table 1.

^b^Raw data of mean IMT scores were multiplied by 100.
